# Culture Growth Phase-Dependent Influence of Extracellular Vesicles Derived from Stem Cells from Human Exfoliated Deciduous Teeth on Oral Mucosa Cells Proliferation in Paracrine Co-Culture with Urethral Epithelium: Implication for Urethral Reconstruction

**DOI:** 10.3390/ijms27010314

**Published:** 2025-12-27

**Authors:** Tsuyoshi Kawaharada, Daisuke Watanabe, Kazuki Yanagida, Kashia Goto, Ailing Hu, Yuhei Segawa, Madoka Higuchi, Masayuki Shinchi, Akio Horiguchi, Tatsuya Takagi, Akio Mizushima

**Affiliations:** 1 Department of Molecular and Cellular Therapeutics, Juntendo University Graduate School of Medicine, Tokyo 113-8421, Japan; 2 Department of Palliative Medicine, Juntendo University Graduate School of Medicine, Tokyo 113-8421, Japan; 3 Department of Urology, Koto Hospital, Tokyo 136-0072, Japan; 4 Division of Reconstruction Center for Trauma, Burn and Tactical Medicine, National Defense Medical College Hospital, Saitama 359-8513, Japan; 5 Department of Urology, National Defense Medical College, Saitama 359-8513, Japan

**Keywords:** cell culture growth phase, extracellular vesicles, stem cells from human exfoliated deciduous teeth (SHED), oral mucosa fibroblasts, indirect co-culture, urethral reconstruction, microRNA, oral mucosal engraftment, urethral stricture, regenerative medicine

## Abstract

Urethral stricture is a disease of fibrotic narrowing that compromises the urethral mucosa and spongiosum. Oral mucosal graft urethroplasty delivers excellent outcomes in complex cases, yet its procedural demands restrict availability beyond specialized centers. Endoscopic transplantation of oral mucosa has been proposed; while feasibility is shown, clinical efficacy remains suboptimal. We asked whether extracellular vesicles from stem cells of human exfoliated deciduous teeth (SHED-EVs) promote oral mucosa fibroblast (OMF) growth under urethra-mimetic paracrine conditions and whether culture growth phase tunes EV function. SHED-EVs were collected during logarithmic (SHED-EV-L) or stationary (SHED-EV-S) phases under xeno-free conditions, isolated by a standardized workflow, and characterized by nanoparticle tracking analysis. miRNA cargo was profiled with a human miRNA microarray platform and normalized for comparative analyses. OMF proliferation was quantified in a horizontal indirect co-culture with urethral epithelial cells using incubator-based time-lapse imaging. SHED-EV-L produced a sustained pro-proliferative effect across 24–96 h, whereas SHED-EV-S showed a weaker early effect with a late catch-up; both exceeded vehicle at 96 h. Fibrosis-related miRNA heat maps showed culture growth phase-dependent patterns: SHED-EV-L displayed relatively higher signals for miR-31-3p, miR-146b-3p, several let-7 members, and selected miR-181 isoforms, whereas SHED-EV-S showed a marked relative increase of miR-486-3p; miR-21, miR-99/100, and miR-205 were broadly comparable between phases. These findings indicate that culture growth phase is a practical design lever that orients SHED-EV cargo and function, supporting phase-matched formulations for adjunctive transurethral applications and motivating in vivo validation and manufacturing-oriented quality controls.

## 1. Introduction

Urethral stricture is commonly managed along a therapeutic spectrum ranging from minimally invasive endoscopic procedures, including dilation and direct visual internal urethrotomy (DVIU), to definitive urethroplasty, with oral mucosal urethroplasty widely considered the gold standard for complex or recurrent cases [1,2]. In clinical practice, however, access to urethroplasty is limited by geographic factors, referral patterns, and surgeon availability. Consequently, endoscopic procedures remain widely performed due to their technical simplicity and minimally invasive nature, despite suboptimal long-term efficacy [3,4]. This widespread use presents a therapeutic opportunity: rather than replacing endoscopic management, its clinical efficacy may be enhanced through the incorporation of biological adjuncts that promote accelerated epithelial regeneration and modulate fibrotic remodeling. Notably, preclinical studies in a rabbit model have demonstrated the feasibility of transurethral mucosal regeneration using minced buccal mucosa delivered with fibrin glue [5,6], and accumulating evidence now supports the broader clinical potential of transurethral oral mucosal grafting. In this context, soluble biological factors that enhance graft incorporation, promote mucosal proliferation, and attenuate early fibrogenic responses have garnered considerable attention.

Extracellular vesicles (EVs) derived from mesenchymal stromal/stem cells (MSCs) have emerged as promising cell-free biological adjuncts for tissue repair. Through the delivery of microRNAs (miRNAs) and other bioactive cargo, MSC-derived EVs can modulate inflammation, fibroblast behavior, and epithelial dynamics. Among various MSC sources, stem cells from human exfoliated deciduous teeth (SHED) represent a particularly attractive option, as SHED-derived EVs contain pro-epithelial and anti-fibrotic miRNA signatures and can be obtained with comparatively high yields. In our previous study [7], we profiled EV-associated miRNAs across different MSC tissue origins and culture growth phases, revealing phase-dependent variations in regulatory miRNAs relevant to mucosal repair, with particularly robust signals observed in SHED. These findings prompted a focused functional evaluation of SHED-derived EVs harvested during the logarithmic and stationary growth phases, with the hypothesis that they may enhance the regenerative potential of transurethral oral mucosal grafting by promoting graft integration and attenuating fibrotic responses in urethral stricture disease.

We herein investigated whether phase-dependent SHED-EV preparations exhibit functional properties in transurethral oral mucosal grafting. Specifically, we tested whether SHED-EVs harvested in the logarithmic versus stationary phases differentially modulate oral mucosa fibroblast (OMF) proliferation in a horizontal, indirect paracrine co-culture system with urethral epithelial cells (UECs), with OMF proliferation quantified by incubator-based live-cell imaging while UECs provided a urethra-mimetic epithelial microenvironment. This hypothesis-generating study aimed to determine whether culture growth phase-specific EVs differentially modulate stromal and epithelial behaviors critical to early mucosal repair, thereby informing EV manufacturing and delivery strategies for future adjunctive applications in transurethral urethral reconstruction.

## 2. Results

### 2.1. Characterization of SHED-Derived Extracellular Vesicles

**Figure 1:**
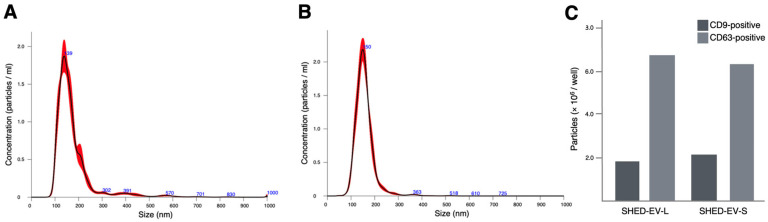
Size distributions of SHED-derived extracellular vesicles by nanoparticle tracking analysis. (**A**) SHED-EV-L. Representative particle size distribution for EVs collected during the logarithmic growth phase. The mean hydrodynamic diameter was 175.0 ± 1.4 nm, the median (D50) was 152.6 ± 1.8 nm, and the mode was 135.9 ± 6.2 nm. Percentile diameters were D10 114.0 ± 1.8 nm and D90 237.8 ± 4.4 nm. Particle concentration was 1.64 × 10^9^ ± 0.06 × 10^9^ particles/mL. (**B**) SHED-EV-S. Representative particle size distribution for EVs collected during the stationary growth phase. The mean hydrodynamic diameter was 157.1 ± 0.9 nm, the median (D50) was 151.4 ± 1.0 nm, and the mode was 150.5 ± 2.9 nm. Percentile diameters were D10 116.1 ± 1.0 nm and D90 197.3 ± 1.9 nm. Particle concentration was 1.54 × 10^9^ ± 0.06 × 10^9^ particles/mL. (**C**) Surface marker profiling of SHED-EV-L and SHED-EV-S by an optical-disc-based ExoCounter assay using CD9–CD9 and CD63–CD63 antibody pairs. Bars show the number of CD9-positive and CD63-positive particles per well for SHED-EV-L and SHED-EV-S (×10^6^/well).

Nanoparticle tracking analysis (NTA) confirmed EV-sized particles for both preparations. For SHED-EV-L, the mean particle size was 175.0 ± 1.4 nm (Figure 1A); percentile diameters were D10 114.0 ± 1.8 nm, D50 (median) 152.6 ± 1.8 nm, and D90 237.8 ± 4.4 nm; the mode was 135.9 ± 6.2 nm; and the size dispersion (SD) was 87.4 ± 2.5 nm. Particle concentration was 1.64 × 10^9^ ± 0.06 × 10^9^ particles/mL. For SHED-EV-S, the mean particle size was 157.1 ± 0.9 nm (Figure 1B), with D10 116.1 ± 1.0 nm, D50 (median) 151.4 ± 1.0 nm, and D90 197.3 ± 1.9 nm; the mode was 150.5 ± 2.9 nm; SD was 43.0 ± 0.8 nm; and the concentration measured 1.54 × 10^9^ ± 0.06 × 10^9^ particles/mL. The detailed NanoSight outputs for SHED-EV-L and SHED-EV-S, including per-run settings and replicate averages, are summarized in Table 1. An optical disc-based ExoCounter assay confirmed the presence of small EVs expressing canonical tetraspanins. In an exploratory run, SHED-EV-L yielded 1.8 × 10^6^ CD9-positive and 6.6 × 10^6^ CD63-positive particles per well, whereas SHED-EV-S yielded 2.1 × 10^6^ and 6.2 × 10^6^ particles per well, respectively. These values were interpreted qualitatively as evidence of CD9^+^/CD63^+^ small EV populations rather than for precise quantification (Figure 1C).

### 2.2. Fibrosis-Related miRNA Cargo of SHED-EVs in Logarithmic and Stationary Phases

**Figure 2:**
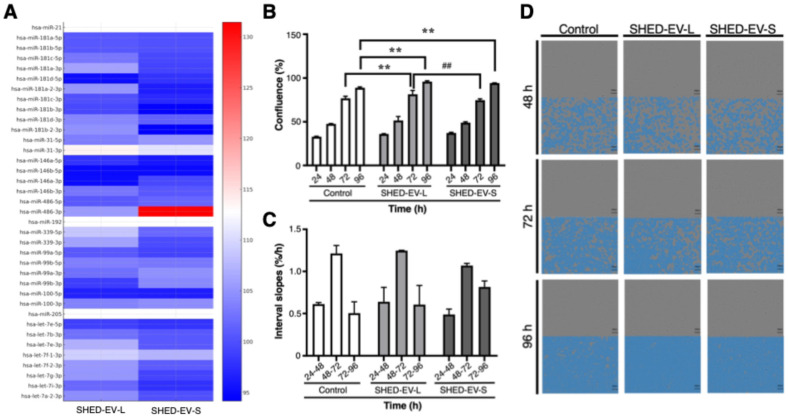
Phase-dependent SHED-EV signatures and OMF proliferation in a urethra-mimetic paracrine model. (**A**) Focused heat map of miRNAs linked to epithelial regeneration and fibrosis modulation (miR-21, miR-181 family, miR-31, miR-146 family, miR-486, miR-192, miR-339, miR-99/100, miR-205, let-7 family) in SHED-EVs collected during the logarithmic growth phase (SHED-EV-L) or the stationary phase (SHED-EV-S). Signals were background-corrected, globally normalized, and median-centered; red indicates higher and blue lower expression relative to the median. (**B**–**D**) Proliferative responses of human oral mucosa fibroblasts (OMFs) indirectly co-cultured with urethral epithelial cells (UECs) in a horizontal plate with laterally linked chambers separated by 1.2 µm porous barriers (no direct contact). EVs were added at doses normalized by nanoparticle tracking analysis. Time-lapse phase-contrast images were acquired in the incubator every 60 min and confluence was quantified by automated segmentation. Data are mean ± SEM (standard error of the mean), n = 3 independent wells per group. (**B**) Confluence at 24, 48, 72, and 96 h for vehicle control, EV-L, and EV-S. Statistical analysis: two-way ANOVA followed by Tukey’s multiple-comparison test (GraphPad Prism 9, San Diego, CA, USA); significance threshold *p* < 0.05. Symbols: ** *p* < 0.01 vs. control; ## *p* < 0.01 vs. SHED-EV-L at the same time point. (**C**) Interval growth rates expressed as confluence slopes over 24–48, 48–72, and 72–96 h (%/h). (**D**) Representative fields at 48, 72, and 96 h for each group, captured at identical magnification; segmentation masks are shown in blue.

Targeted profiling of fibrosis-related miRNAs showed culture growth phase-dependent patterns (Figure 2A). The heat map summarizes relative abundance for miR-21, the miR-181 family, miR-31, the miR-146 family, miR-486, miR-192, miR-339, miR-99/miR-100, miR-205, and the let-7 family in EVs collected during the logarithmic (SHED-EV-L) and stationary (SHED-EV-S) phases. In SHED-EV-L, relative signals appeared higher for miR-31, miR-146b-3p, members of the let-7 family, and several miR-181 members. In SHED-EV-S, miR-21 was comparable to SHED-EV-L, whereas miR-486-3p showed a marked relative increase. Conversely, miR-31-3p was relatively higher in SHED-EV-L, with modest enrichment of selected let-7 and miR-146 family members. Signals for miR-99/miR-100 and miR-205 were broadly similar across phases without a consistent directional shift. Values are median-centered and presented descriptively without formal differential testing. Taken together, the cargo profile differs by culture growth phase, indicating a pattern in which SHED-EV-L is relatively enriched for miRNAs associated with epithelial support, whereas SHED-EV-S shows greater representation of remodeling-associated miRNAs.

### 2.3. Proliferative Responses of OMFs to SHED-EVs in Indirect Co-Culture with UECs

Time-lapse imaging showed a progressive increase in OMF confluence across all groups in the horizontal, non-contact co-culture with UECs (Figure 2B–D). Group differences were tested by two-way ANOVA with Tukey’s post hoc tests at each time point (n = 3 per group; mean ± SEM). Confluence (%) was 33.06 ± 0.51 (control), 36.18 ± 0.44 (SHED-EV-L), and 37.36 ± 0.70 (SHED-EV-S) at 24 h; 47.73 ± 0.32, 51.51 ± 4.55, and 49.07 ± 0.92 at 48 h; and 76.86 ± 2.31, 81.43 ± 4.46, and 74.74 ± 1.52 at 72 h, respectively. By 96 h, values reached 88.97 ± 0.94 (control), 95.95 ± 1.03 (SHED-EV-L), and 94.31 ± 0.29 (SHED-EV-S). No between-group differences were detected at 24 h or 48 h. At 72 h, SHED-EV-L exceeded control (mean difference 4.57%, 95% CI 0.01–9.13; *p* = 0.0497) and also exceeded SHED-EV-S (difference 6.70%, 95% CI 2.14–11.26; *p* = 0.0053). At 96 h, both EV groups were greater than control (SHED-EV-L vs. control: difference 7.00%, 95% CI 2.44–11.56; *p* = 0.0039; SHED-EV-S vs. control: difference 5.37%, 95% CI 0.81–9.93; *p* = 0.0216), whereas SHED-EV-L and SHED-EV-S did not differ (*p* = 0.6173).

Interval growth rates (Figure 2C) reinforced these patterns: during 24–48 h, growth was modestly higher with SHED-EV-L than control (0.639 ± 0.172 vs. 0.611 ± 0.020%/h) and lower with SHED-EV-S (0.488 ± 0.065%/h); during 48–72 h, all groups accelerated (control 1.214 ± 0.095%/h; SHED-EV-L 1.246 ± 0.004%/h; SHED-EV-S 1.070 ± 0.025%/h); and during 72–96 h, SHED-EV-S showed the greatest late-phase increase (0.815 ± 0.071%/h) relative to SHED-EV-L (0.605 ± 0.229%/h) and control (0.505 ± 0.135%/h). (Slope comparisons are descriptive only.) Collectively, SHED-EV-L produced a consistent, durable pro-proliferative effect across 96 h, whereas SHED-EV-S displayed a weaker early effect with a late catch-up, indicating a growth-phase-dependent influence of SHED-EVs on OMF proliferation under paracrine conditions.

## 3. Discussion

In this study, we evaluated SHED-derived EVs using a horizontal, noncontact paracrine coculture system pairing UECs with OMFs, which enabled continuous, incubator-based monitoring of OMF proliferation. EV preparations satisfied vesicle-scale criteria by nanoparticle tracking analysis. Fibrosis-focused miRNA profiling revealed distinct growth phase-dependent signatures: EVs harvested during the logarithmic phase (SHED-EV-L) exhibited relative enrichment of miRNAs associated with epithelialization and anti-fibrotic signaling, whereas EVs harvested during the stationary phase (SHED-EV-S) displayed elevated levels of miRNAs linked to matrix remodeling pathways. Functionally, SHED-EV-L induced sustained pro-proliferative effects over 24–96 h, whereas SHED-EV-S exerted modest early effects with delayed acceleration between 72 and 96 h. The concordance between miRNA cargo profiles and these temporally resolved proliferative responses represents the principal finding of this study.

OMFs contribute to graft integration and wound closure by remodeling the extracellular matrix and secreting paracrine factors, including keratinocyte growth factor (KGF) and hepatocyte growth factor (HGF), that modulate epithelial behavior [8]. These characteristics align with the inherently rapid, low-scarring healing observed in oral mucosa [8]. Because epithelial–stromal crosstalk critically shapes early tissue repair, noncontact paracrine models are well suited to dissecting EV-mediated effects. The UniWells horizontal indirect coculture platform preserves paracrine interactions between OMFs and UECs via lateral microchannels separated by size-selective filters, while live-cell incubator imaging provides unbiased, temporally resolved quantification of cell proliferation and wound closure. Within this urethra-mimetic in vitro system, the effects of SHED-EVs on OMF proliferation may be partially attributable to their miRNA cargo.

For fibrosis-focused analysis, we examined a predefined miRNA panel comprising miR-21 [9,10,11], the miR-181 family [12], miR-31 [13,14,15], the miR-146 family [16], miR-486 [17], miR-192 [18], miR-339 [19], the miR-99 family [20,21,22], miR-205 [23], and the let-7 family [24,25,26]. EVs harvested during the logarithmic phase exhibited elevated levels of miR-31, let-7 family members, miR-146a, and miR-29. These miRNAs have been implicated in promoting epithelial coverage and migration, attenuating NF-κB-driven inflammation, and suppressing TGF-β/SMAD signaling with downstream inhibition of COL1A1, COL3A1, and ACTA2 expression. In the urethral epithelial–stromal paracrine context, such a molecular signature would be expected to condition fibroblasts toward a proliferative yet less myofibroblastic phenotype while supporting epithelial resurfacing, which is consistent with the sustained increase in OMF confluence observed with SHED-EV-L treatment over 24 to 96 h.

Collectively, these data suggest that culture growth phase serves as a practical determinant to bias SHED-EV molecular signaling toward either an epithelial-supportive, anti-fibrotic program or a matrix remodeling-oriented program. However, these associations remain correlative, and miRNA gain- and loss-of-function experiments will be required to establish causality.

Clinically, the most immediate translational opportunity lies in facilitating transurethral oral mucosal engraftment and enhancing early re-epithelialization following endoscopic procedures. The acute postoperative period encompasses the immediate interval after mucosal injury and resurfacing procedures, such as DVIU combined with transurethral oral mucosal grafting. During this critical window, the urethral lumen remains partially denuded and exposed to urine, a provisional fibrin matrix is forming, inflammatory mediators are elevated, and fibroblasts undergo activation. Rapid epithelial coverage within the initial days is essential to seal the wound surface and to limit myofibroblast proliferation and collagen deposition that ultimately drive restenosis. A similar acute milieu arises after iatrogenic urethral trauma, for example catheter-related injury, where prompt resurfacing likewise mitigates fibrotic remodeling. Against this backdrop, the present data support a temporally staged approach: when the clinical priority is rapid epithelial closure during the immediate post-injury period, SHED-EV-L may be preferred; when subsequent matrix organization and remodeling become the therapeutic focus, SHED-EV-S may be incorporated as a sequential or combination regimen. Practical delivery modalities align with standard cystoscopic workflows and include intralesional or submucosal injection of EV-laden mucoadhesive gels immediately following dilation or incision, transurethral instillation of shear-thinning or in situ-gelling EV formulations to coat the treated segment, and short-term placement of EV-releasing catheters to provide sustained local exposure during the early healing phase.

In our previous proposal [7], two complementary translational strategies were outlined: supporting oral mucosal graft urethroplasty at tertiary referral centers and augmenting routine endoscopic management through transurethral delivery. The current findings are consistent with this framework and indicate that selecting EV preparations based on culture growth phase and pairing them with practical delivery platforms may effectively target the biological determinants of restenosis—namely, incomplete re-epithelialization and excessive fibrosis—while maintaining a technically straightforward and scalable procedural pathway.

Despite these findings, several limitations warrant acknowledgment. First, the present system isolates paracrine signaling in a horizontal, noncontact coculture model and does not incorporate direct epithelial–stromal contact or immune cell components; validation in triculture and contact coculture systems is warranted. Second, functional assays were conducted with n = 3 per group, which constrains statistical power; future studies will expand biological EV replicates and employ prespecified primary endpoints with mixed-effects statistical models. Third, EVs were isolated using a polymer-based precipitation method, which may co-enrich non-vesicular contaminants; orthogonal purification strategies, including size-exclusion chromatography or density-gradient ultracentrifugation, will be implemented to validate key findings. Fourth, the miRNA profiling provides correlative evidence; establishing causality will require miRNA gain- and loss-of-function experiments using antagomirs or mimics, coupled with pathway-specific reporters in the same coculture context. Fifth, all EV isolations and functional characterizations were performed under standardized in vitro conditions that may not fully recapitulate the complex urethral microenvironment. Fibroblast responses in this study were captured only as confluence-based proliferation in a urethra-mimetic indirect co-culture; we did not include additional functional assays such as scratch-closure assays, myofibroblast differentiation markers, matrix deposition, or migration. These in-depth in vitro assays, together with in vivo validation models, will be essential in future work to more fully define the effects of SHED-EVs on fibroblast behavior and urethral healing. Sixth, we employed confluence-based operational criteria to standardize EV harvest timing; accordingly, the logarithmic and stationary designations reflect preconfluent expansion and postconfluent plateau phases rather than precise cell-cycle states. Seventh, EV dosing used stock suspensions rather than particle-matched inputs; the nominal particle loads per device were approximately 2.46 × 10^9^ for EV-L and 2.31 × 10^9^ for EV-S, corresponding to a 7% difference based on NTA. Although this difference is modest and unlikely to account for the magnitude or direction of the effects, future experiments will normalize by particle number and/or EV protein and will include dilution series to exclude dose contributions. Until these additional datasets become available, the present conclusions should be regarded as hypothesis-generating and require further validation. Eighth, we did not include a comparison group in which OMFs were cultured in the same device in the absence of UECs. As a result, the present data cannot disentangle the specific contribution of the epithelial compartment to the observed EV effects on OMF proliferation. Future experiments will incorporate fibroblast-only and epithelium-free conditions, as well as alternative epithelial sources, to formally quantify the regulatory role of urethral epithelium in this model.

## 4. Materials and Methods

### 4.1. Cell Sources and Culture Conditions

Stem cells from human exfoliated deciduous teeth (SHED; Summit Pharmaceuticals International, Tokyo, Japan; ATCC^®^ CRL-3485™) were expanded at 37 °C in a humidified 5% CO_2_ atmosphere. SHED were seeded at 1500 cells/cm^2^ and cultured in a xeno-free medium supplemented with human serum (Takara Bio, Kusatsu, Japan; Cat. Y30001) until reaching ~5000 cells/cm^2^ over ~36 h, corresponding to the logarithmic growth phase. Immediately prior to extracellular vesicle (EV) collection, cultures were switched to an animal-origin-free (AOF) medium (Rohto Pharmaceutical, Osaka, Japan), as detailed below.

### 4.2. Definition of Growth Phases and Conditioned Medium Collection

EV harvest followed objective culture features rather than elapsed time, with a fixed conditioned-medium (CM) collection window in AOF medium. Logarithmic (SHED-EV-L) cultures were defined as pre-confluent monolayers (40–70% confluence) showing ongoing expansion over the preceding 24 h (≥30% increase in viable cell counts per well) and viability ≥90%. Stationary (SHED-EV-S) cultures were defined as monolayers at ≥95% confluence maintained for 24–48 h with plateaued growth (≤10% change in viable counts over 24 h) and viability ≥90%. Once criteria were met, cultures were maintained in AOF and CM was collected over a 24 h window.

### 4.3. Extracellular Vesicle Isolation and Particle Characterization

CM collected as in Section 2.2 was sequentially clarified by low-speed centrifugation (300× *g* for 10 min; 2000× *g* for 10 min) and 0.22 µm filtration. EVs were isolated using a polymer-based precipitation method (EXO Isolation Reagent Kit, Dojindo, Kumamoto, Japan; Cat. EX10) according to the manufacturer’s instructions. Pellets were resuspended in sterile, 0.22 µm-filtered phosphate-buffered saline (PBS), gently mixed on ice, and aliquoted to avoid repeated freeze–thaw cycles.

Particle concentration and size distribution were determined by nanoparticle tracking analysis (NTA; NanoSight, Malvern Instruments, Worcestershire, UK). For each biological replicate, three 60 s videos were acquired at room temperature with identical camera level and detection threshold across all conditions. Instrument performance was verified at each session using the vendor’s standard operating procedures. EV dosing in functional assays was normalized by particle number as quantified by NTA; exact doses for each experiment are reported in the figure legends.

To complement NTA-based measurements, surface tetraspanin-positive small EVs were quantified using an optical disc-based ExoCounter assay as previously described [27]. Briefly, EV samples were applied to antibody-coated discs targeting CD9 or CD63 and incubated to allow binding of small EVs to capture nanobeads. After washing to remove unbound material, the discs were scanned on the ExoCounter instrument (JVC KENWOOD, Kanagawa, Japan; assay kits BX-EAK1JA and BX-EAK2JA), and signals were converted to CD9-positive or CD63-positive particle counts per well according to the manufacturer’s instructions. For the present study, one exploratory measurement was performed per condition; therefore, ExoCounter data are reported as single values without measures of dispersion and are interpreted qualitatively as confirmation of CD9^+^/CD63^+^ small EV populations.

### 4.4. EV Small RNA Extraction and Microarray-Based miRNA Profiling

Total RNA including small RNAs was isolated from EV aliquots with a phenol-free, column-based kit compatible with low-input preparations. RNA yield was quantified by fluorescence-based assays optimized for small RNAs. Genome-wide miRNA expression profiling used the human 3D-Gene microarray platform (Toray Industries, Tokyo, Japan) under xeno-free and serum-free EV collection conditions. Labeling, hybridization, washing, and scanning followed the vendor’s protocols. Raw intensity files were exported for downstream processing, background was corrected, and global median normalization was applied across arrays. Normalized values were log_2_-transformed. For fibrosis-focused visualization, a predefined panel was assembled comprising miR-21, miR-181 family, miR-31, miR-146 family, miR-486, miR-192, miR-339, miR-99, miR-100, miR-205, and the let-7 family (Table 2 for functional summaries and primary references). Heat maps display median-centered, normalized values, with red indicating higher and blue indicating lower expression relative to the median.

### 4.5. Cell Proliferation Test (Horizontal Indirect Co-Culture with Real-Time Imaging)

**Figure 3:**
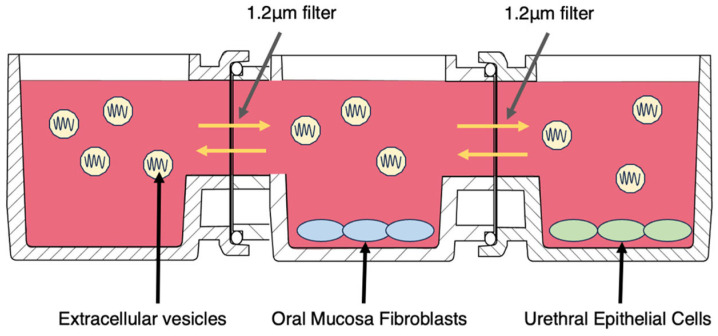
Horizontal indirect co-culture of oral mucosa fibroblasts with urethral epithelial cells and SHED-derived extracellular vesicles. Schematic of the horizontal indirect co-culture used for paracrine assays. Three chambers are linked in series by lateral microchannels separated by 1.2 µm porous barriers that block cell migration while permitting diffusion of soluble factors and extracellular vesicles (EVs). SHED-EV-L or SHED-EV-S are added to the left reservoir; oral mucosa fibroblasts (OMFs) are seeded in the central chamber; urethral epithelial cells (UECs) are seeded in the right chamber. A connector allows three or more modules to be linked. Arrows indicate bidirectional lateral diffusion and the absence of direct OMF–UEC contact. Cultures were imaged in the incubator every 60 min for up to 96 h; OMF proliferation was quantified as confluence and interval slopes.

Primary human oral mucosa fibroblasts (OMFs; hOMF100, CellResearch Corporation; distributor Cosmo Bio, Tokyo, Japan; received at P3) and human urethral epithelial cells (UECs; HUEpC, Cell Applications, Cat. 9310-05a) were expanded according to the suppliers’ instructions at 37 °C with 5% CO_2_ and were used at passages ≤ 6. A horizontal indirect co-culture device (UniWells; Ginrei Lab) [28] was assembled in a three-chamber layout linked in series by lateral microchannels separated with 1.2-µm porous barriers. Chambers were assigned as follows: left, EV/vehicle reservoir; middle, OMFs; right, UECs (Figure 3).

Cells were thawed, counted, and diluted in their dedicated growth media. UECs and OMFs were each seeded at 5000 cells/cm^2^ into their respective chambers (UECs to the right chamber in UEC medium; OMFs to the middle chamber in OMF medium). After attachment, each chamber was gently brought to a final volume of 1.5 mL, ensuring that the channels and barrier faces were fully covered and that liquid levels were identical across chambers to permit lateral diffusion and avoid convective flow. SHED-derived EVs collected in the logarithmic (SHED-EV-L) or stationary (SHED-EV-S) culture phases were quantified by nanoparticle tracking analysis and stored in PBS as stock suspensions (see Section 2.1). For dosing, the left chamber received 1.5 mL of either PBS alone (Control) or the EV stock. Thus, the approximate particle inputs per device were 2.46 × 10^9^ ± 0.09 × 10^9^ particles for SHED-EV-L and 2.31 × 10^9^ ± 0.09 × 10^9^ particles for SHED-EV-S, based on NTA-derived concentrations (1.64 × 10^9^ ± 0.06 × 10^9^ and 1.54 × 10^9^ ± 0.06 × 10^9^ particles/mL, respectively). Because the two preparations differed slightly in concentration and size distribution, comparisons in this study reflect phase-associated EV preparations rather than strictly dose-matched vesicle counts.

Devices were placed on an incubator-compatible live-cell monitoring system (CM30; EVIDENT). Phase-contrast images of the OMF chamber were acquired every 60 min from 24 h to 96 h at fixed stage positions. OMF proliferation was quantified as (i) confluence (% area) obtained by automated segmentation and (ii) interval growth rates (slopes of confluence between 24–48, 48–72, and 72–96 h). Fields of view and technical replicates were preset and kept identical across groups.

### 4.6. Statistical Analysis

All quantitative data are presented as mean ± SEM (standard error of the mean). Unless otherwise specified, n denotes biological replicates (independent wells seeded from the same cell lot; EVs from the same batch). Multiple fields per well were averaged to yield one value per well at each time point. No data were excluded a priori and no imputation was performed. For time–confluence measurements at 24, 48, 72, and 96 h, a two-way ANOVA with factors group (Control, SHED-EV-L, SHED-EV-S) and time was used, followed by Tukey’s post hoc test for pairwise comparisons at matched time points. All tests were two-sided with α = 0.05; *p* < 0.05 was considered statistically significant. Analyses and plotting were performed in GraphPad Prism 9 (GraphPad Software, San Diego, CA, USA). Interval growth rates (24–48, 48–72, 72–96 h) are presented descriptively as mean ± SEM to illustrate temporal patterns and were not subjected to formal hypothesis testing. For miRNA heat maps, array intensities were background-corrected, globally normalized, and median-centered; unless otherwise stated, these displays are descriptive.

## 5. Conclusions

In conclusion, the culture growth phase is a practical design lever that orients SHED-EV miRNA cargo and aligns with distinct proliferative effects on oral mucosa fibroblasts in a urethra-mimetic paracrine co-culture. EVs collected in the logarithmic phase favored an epithelial-supportive, anti-fibrotic profile and yielded sustained gains in confluence, whereas stationary-phase EVs were more consistent with a remodeling-oriented profile and a later catch-up. These findings support the development of phase-matched EV formulations, together with simple cystoscopic delivery formats, for adjunctive use in transurethral oral mucosal engraftment and endoscopic management of urethral strictures. This work is hypothesis generating and motivates in vivo validation and manufacturing-oriented quality controls that include miRNA markers in addition to particle metrics.

## Figures and Tables

**Table 1:** Detailed results of nanoparticle tracking analysis of SHED-EVs.

Parameters	SHED-EV-L (nm, Mean ± SEM)	SHED-EV-S (nm, Mean ± SEM)
**Mean**	175.0 ± 1.4	157.1 ± 0.9
**Mode**	135.9 ± 6.2	150.5 ± 2.9
**SD**	87.4 ± 2.5	43.0 ± 0.8
**D10**	114.0 ± 1.8	116.1 ± 1.0
**D50 (Median)**	152.6 ± 1.8	151.4 ± 1.0
**D90**	237.8 ± 4.4	197.3 ± 1.9
**Particle concentration** **(particles/mL)**	1.64 × 10^9^ ± 0.06 × 10^9^	1.54 × 10^9^ ± 0.06 × 10^9^

EV concentrations (particles/mL) were quantified using nanoparticle tracking analysis (NanoSight, Malvern Instruments, Worcestershire, UK). Abbreviations: EV = extracellular vesicle; SHED = stem cells from human exfoliated deciduous teeth; Mode = peak diameter (highest concentration; D10, D50, and D90 denote the 10th, 50th (median), and 90th percentile diameters, respectively).

**Table 2:** Functional Overview of Selected miRNAs Related to Mucosal Regeneration and Fibrosis Modulation.

miRNA	Primary Function	Key Target/Pathways	Implications for Mucosal Grafting	References
**miR-21**	Anti-apoptotic, pro-survival, anti-inflammatory	PTEN/AKT, TGF-β	Facilitates early tissue adaptation and helps suppress fibrotic responses	[9,10,11]
**miR-181**	Anti-inflammatory/anti-fibrotic tuning; restrains EMT	NF-κB axis (via IKK signaling), TGF-β/SMAD signaling	May temper early fibro-inflammation and myofibroblast conversion, supporting stable re-epithelialization and reducing scar formation	[12]
**miR-31**	Promotes proliferation and migration	RhoA, FZD3, Wnt/β-catenin	Accelerates initial epithelial closure and graft integration	[13,14,15]
**miR-146**	Anti-inflammatory, antioxidative, immune-suppressive	TRAF6, IRAK1	Resolves chronic inflammation, reduces oxidative stress, and facilitates transition from inflammatory to proliferative phase during wound healing	[16]
**miR-486**	Anti-fibrotic; pro-re-epithelialization	IGF1–PI3K/AKT, SMAD2/3; AKT3 in keratinocyte migration	Could enhance epithelial coverage while limiting collagen I/III deposition and hypertrophic scar-like remodeling	[17]
**miR-192**	Context-dependent regulator of fibrosis	TGF-β/SMAD network; ECM gene programs	Signal is cell-/context-specific in epithelial tissues; when present, may require phase- or dose-aware use to avoid pro-fibrotic drift	[18]
**miR-339**	Epithelial phenotype maintenance; EMT restraint; differentiation control	BCL6 (EMT-linked), Wnt/β-catenin pathway components	May help stabilize epithelial identity and curb EMT-driven contraction during graft take; evidence is indirect and hypothesis-generating	[19]
**miR-99 family** **(miR-99a/miR-100)**	Regulates proliferation and metabolism	mTOR/IGF1R pathway	Maintains controlled epithelial proliferation and energy balance during early repair	[20,21,22]
**miR-205**	Maintains epithelial phenotype, inhibits EMT	ZEB1/2, E-cadherin	Promotes epithelial integrity; downregulation is required for keratinocyte migration during re-epithelialization; potential therapeutic target in chronic wounds	[23]
**let-7 family**	Controls cell cycle and differentiation; tumor suppression	RAS, HMGA2	Promotes epithelial stability and suppresses oncogenic transformation in vitro and in vivo	[24,25,26]

## Data Availability

The data presented in this study are available in this article.

## Abbreviations

The following abbreviations are used in this manuscript:

ANOVA	Analysis of variance
AOF	Animal-origin-free (medium)
CI	Confidence interval
CM	Conditioned medium
DVIU	Direct visual internal urethrotomy
ECM	Extracellular matrix
EMT	Epithelial–mesenchymal transition
EV	Extracellular vesicle
HGF	Hepatocyte growth factor
HMGA2	High mobility group AT-hook 2
IGF1R	Insulin-like growth factor-1 receptor
KGF	Keratinocyte growth factor
miRNA	MicroRNA
MSC	Mesenchymal stromal/stem cell
mTOR	Mechanistic target of rapamycin
NF-κB	Nuclear factor kappa-B
NTA	Nanoparticle tracking analysis
OMF	Oral mucosa fibroblast
OMG	Oral mucosal graft
PBS	Phosphate-buffered saline
RAS	RAS proto-oncogene family (family of small GTPases)
SD	Standard deviation
SEM	Standard error of the mean
SHED	Stem cells from human exfoliated deciduous teeth
TGF-β	Transforming growth factor-β
UEC	Urethral epithelial cell
